# Supplementary material for: Optogenetic manipulation of cell migration with high spatiotemporal resolution using lattice lightsheet microscopy
Source: Commun Biol. 2022 Aug 26;5:879. doi: 10.1038/s42003-022-03835-6 (PMC9418249; doi:10.1038/s42003-022-03835-6)
Supplement: Supplementary file 3 — Description of Additional Supplementary Files [file 42003_2022_3835_MOESM3_ESM.pdf]

Description of Additional Supplementary Files

Supplementary Movie 1

Time-lapse XY’ MIP images of CRY2olig-mRuby3 expressed cells with various activation energies (0.5, 1, 2, and 4 nW). The interval between each stack is 3 seconds. The first stimulation starts at stack 20. The selected time point MIP images are shown in Fig. 1e. The video playback speed is 10 Hz.

Supplementary Movie 2

Time-lapse XY’ MIP images of CRY2olig-mRuby3 expressed cells stimulated with different wavelengths (405, 445, 488, and 514 nm) at 1 nW. The interval between each stack is 3 seconds. The first stimulation starts at stack 20. The selected time point MIP images are shown in Fig. S3. The video playback speed is 10 Hz.

Supplementary Movie 3

Time-lapse images of optically induced clustering of CRY2oligo-mRuby3 in XY' Y'Z' XZ' planes by Bessel fan activation. The depth of the oligomerization is color-coded. The interval between each stack is 3 seconds. And the stimulation starts at stack 20. The selected images at different time points are displayed in Fig. 2b. The video playback speed is 10 Hz.

Supplementary Movie 4

Time-lapse images of optically induced clustering of CRY2oligo-mRuby3 in XY' Y'Z' XZ' planes by shifted Bessel beam activation. The depth of the oligomerization is color-coded. The interval between each stack is 3 seconds. The first stimulation starts at stack 20. The selected images at different time points are displayed in Fig. 2b. The video playback speed is 10 Hz.

Supplementary Movie 5

Time-lapse images of optically induced clustering of CRY2oligo-mRuby3 in XY' Y'Z' XZ' planes by single Bessel beam activation. And the depth of the oligomerization is color-coded. The interval between each stack is 3 seconds. The first stimulation starts at stack 20. The selected images at different time points are displayed in Fig. 2c. The video playback speed is 10 Hz.

Supplementary Movie 6

Time-lapse XY MIP images of subcellular activation of the cell expressing CRY2mCherryiSH-p2a-CIBN-CAAX by Bessel fan activation, where the cyan color is the activation site. The interval between each stack is 3 seconds. The interval between each stack is 3 seconds. The

first stimulation starts at stack 30 and restarts at stack 102 (rest time = 30min). The selected images at different time points are displayed in Fig. S4. The video playback speed is 10 Hz.

Supplementary Movie 7

Time-lapse XY' MIP images of optically induced membrane ruffling of cell expressing F-tractin-mCherry-p2a-CRY2iSH-p2a-CIBN-CAAX by single Bessel beam activation, where the cross is the activation site. The interval between each stack is 6 seconds. The stimulation starts at stack 30. The selected images at different time points are shown in Fig. 3a. The video playback speed is 10 Hz.

Supplementary Movie 8

Time-lapse XY' MIP images of optically induced cell migration of cell expressing EGFR-CRY2Olig-mApplex3 by Bessel fan activation, where the cyan line is the activation site. The interval between each stack is 6 seconds. And the stimulation starts at stack 20. The selected images at different time points are shown in Fig. 3b. The video playback speed is 10 Hz.

Supplementary Movie 9

Raw 3D MIP image of optically induced cell migration of cell expressing EGFR-CRY2Olig-mApplex3 by Bessel fan activation. The cell was activated at T=0, Stack=60.The total stack number is 463 for about 6 hr observation. During the acquisition, the migrating cell was positioned at the imaging center of the field of view.

Supplementary Movie 10

The selected 18 XY' MIP images of cell migration for a cell expressing EGFR-CRY2OligI-mApplex3 activated by the Bessel fan. The cyan line represents the activation site. Different color codes are used to represent the contours of the cells at different time points. Selected time points are shown in Figure 4.

Supplementary Movie 11

The Raw 2D images used to reconstruct 3D volumetric image in the Bessel fan photoactivation experiment, where CRY2oligo-mRuby3 molecules are stimulated by 488 nm across the whole cell. 488 nm can excite the activated CRY2oligo-mRuby3 clusters with low efficiency, where the weak fluoresce signals reveal the contributions of main and side lobes of the illuminated Bessel beam forming a stipe of a width of 2 μm (left) as opposed to a lattice sheet imaging by 560 nm (right). Note that the residual electronic signal in the sCMOS leaking from the 560 nm channel was observed at the 488 nm channel.

Supplementary Movie 12

The Raw 2D images used to reconstruct 3D volumetric image of a single Bessel beam photoactivation experiment. Only a weak fluorescent signal was observed at the selected plane (z=55), where the cell was illuminated by 488 nm (left). In comparison, a lattice light-sheet imaging at 560 nm was used to record the fluorescence signals of the cell (right).
